# Analysis of the hump phenomenon and needle defect states formed by driving stress in the oxide semiconductor

**DOI:** 10.1038/s41598-019-48552-z

**Published:** 2019-08-19

**Authors:** Hyeon-Jun Lee, Katsumi Abe, Hee Yeon Noh, June-Seo Kim, Hyunki Lee, Myoung-Jae Lee

**Affiliations:** 1 Intelligent Devices & Systems Research Group, Institute of Convergence, DGIST, Daegu, 42988 Korea; 2 Silvaco Japan Co. Ltd., Nakagyo-ku, Kyoto 604-8152 Japan; 3 Convergence Research Center for Collaborative Robots, Institute of Convergence, DGIST, Daegu, 42988 Korea

**Keywords:** Electrical and electronic engineering, Electronic and spintronic devices

## Abstract

The reduction in current ability accompanied by the hump phenomenon in oxide semiconductor thin-film transistors to which high DC voltages and AC drive voltages are applied has not been studied extensively, although it is a significant bottleneck in the manufacture of integrated circuits. Here, we report on the origin of the hump and current drop in reliability tests caused by the degradation in the oxide semiconductor during a circuit driving test. The hump phenomenon and current drop according to two different driving stresses were verified. Through a numerical computational simulation, we confirmed that this issue can be caused by an additional “*needle*”, a shallow (~0.2 eV) and narrow (<0.1 eV), defect state near the conduction band minimum (CBM). This is also discussed in terms of the dual current path caused by leakage current in the channel edge.

## Introduction

Amorphous InGaZnO (*a*-IGZO) is a promising material in high performance displays owing to its high field-effect-mobility and extremely low leakage current compared to those of the conventional amorphous silicon (a-Si:H) thin film transistors (TFTs). Recently, this material has been successfully employed in pixel driver circuitry for commercial display applications^1^. However, the instability in this oxide semiconductor with bias, temperature, and illumination during long-term operation remains a critical issue^2,3^. When an alternative high gate and drain bias are applied in the transistors, the electrical degradation by the high voltage and alternative pulse signal causes the deterioration of the driving signal. The driving long-term stability in the oxide semiconductor has been studied from various perspectives: charge trapping^4^, defect creation^5^, ambient effect^6^, impact ionization^3^, and hot carrier injection^7^. Among these causes, the common phenomenon observed is the “*hump*” characteristic in the current–voltage (I–V)^8–10^ or capacitance–voltage (C–V)^11^ measurement.

Hump generation is a characteristically visualized typical error phenomenon known as abnormal current path. Analysis of the causes of hump by two or more current paths^12^ has been reported in many studies on metal-oxide-semiconductor field-effect transistors (MOSFETs)^13^ and low-temperature polycrystalline silicon (LTPS)^12,14^. Apart from the hump phenomenon in silicon-based transistors, the hump in oxide semiconductors has been researched over the past 10 years under various conditions (bias stress conduction) and circumstances (temperature and illumination), such as reduction in current capability, early turned-on in the threshold voltage^8^ region, or bidirectional phenomena^15^. In particular, the hump phenomena, which occur under the driving test including a positive gate bias stress test with temperature^16^ or illumination^8,17^, were interpreted as the back-channel conduction^14^, formation of the defects^15^, edge effects^18^, or parasitic TFTs^12,19^. In existing reports, under conditions that enable increasing of the driving current, such as an increase in the environment temperature and an examination of light during the stress evaluation, it is common to see that a hump is manifested. In addition, in recent research carried out on the abnormal drive of a transistor by the drain current stress^20,21^, a hump phenomenon has been reported. Although there have been reports on the hump effect under the subthreshold operation as an edge effect back-channel conduction or parasitic TFTs, the hump effect has not been fully studied in oxide semiconductors under various driving stress environments. In particular, the hump issue under drain current stress has not been studied in detail yet.

Here, we investigated the abnormal electric hump characteristic in the oxide semiconductor under various stress conditions. Two different types of driving stress were applied: DC constant voltage stress (DCVS) at the gate with drain side (testing for pixel TFTs) and pulsed high-voltage drain bias stress (pulsed-HVDS)/pulsed high-voltage gate bias stress (pulsed-HVGS), testing for integrated circuit TFTs in the gate driver^3^. The hump was observed in both cases and confirmed via electrical measurement and technology computer-aided design (TCAD)^22^ simulation. It was confirmed that the hump is caused not only by an increasing current in the transistor, but also by a transient current caused by pulse-type stress. We determined that this issue can be caused by shallow (~0.2 eV) and narrow (<0.1 eV) defect states near the conduction band minimum (CBM). In particular, we found that the studied hump phenomena are independent of the type of shallow defects, and they heavily depend on the size and width of the defects. In addition, the hump caused by a dual current path from the channel edge current was also discussed through 3D simulation of the device.

## Results

**Figure 1:**
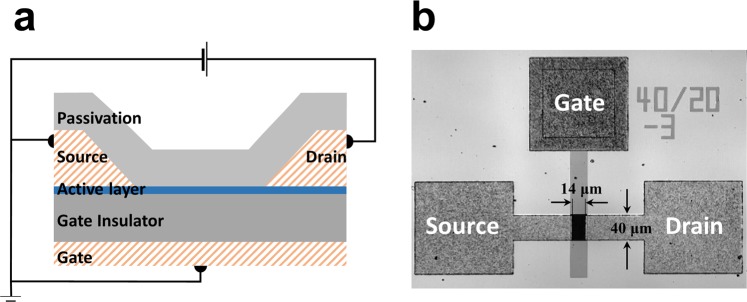
Bottom-gated top-contact structure. (**a**) Schematic cross-sectional view of an *a*-IGZO_x_ driving TFT with a 40-nm thick active layer. The sample was fabricated with the same BCE structure and the active layer is defined by an independent mask. (**b**) Top-view microscope image of the *a*-IGZO_x_ TFT with W = 40 μm and L = 14 μm. The designed overlap between the source/drain electrode and the gate electrode was 3 μm.

Figure 1 shows the schematic cross-sectional view of an in-cell structure and a microscopic image with the driving TFT in this work embedded in a display panel. The samples were stacked on a bottom gate structure, which is commercially applied in the display active matrix driving parts. In a bottom-gate top contact device structure, two different TFT configurations are possible, i.e., back channel etch (BCE)^23^ and etch-stop layer (ESL)^24^. Although the device bias-stress stability is reported to be better for ESL TFTs compared to that for BCE TFTs, the BCE configuration is preferred to the ESL structure in the industry because it allows saving two mask photo-steps. In this work, the samples were prepared with the same BCE structure, but the active BCE process was not utilized in the source/drain half-tone mask step^25^. It was designed by a 3 μm overlap between the source/drain and gate electrode.

### Reduction of current in the hump formed by damage

**Figure 2:**
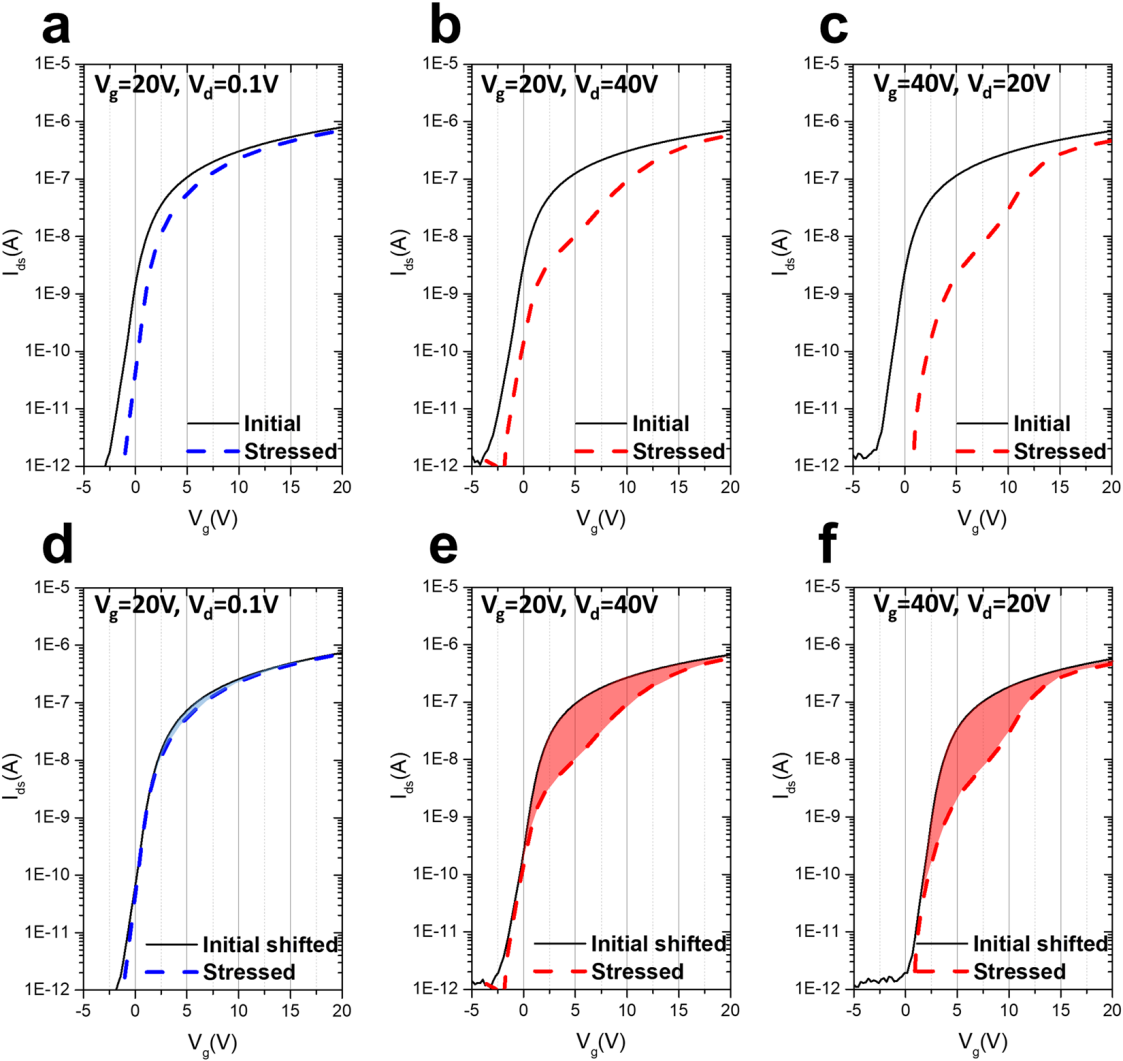
Initial and stressed I–V characteristics under V_ds_ of 0.1 V depending on the constant bias stresses. The constant bias stress was applied at 60 °C for 1.5 h; (**a**) V_g_ = 20 V, V_ds_ = 0.1 V; (**b**) V_gs_ = 20 V, V_ds_ = 40 V; and (**c**) V_gs_ = 40 V, V_ds_ = 20 V. The black solid line indicates the initial I–V characteristics. The blue and red dashed lines are the I–V sweep signals after the stress. To clarify the current drop at the threshold voltage region by hump depending on each stress, the I–V characteristics were compared through the initial I–V shifted to the stressed I–V. (**d**–**f**) Clearly show the current drop and hump behavior in (**a**–**c**), respectively. The gap between the initial shifted and stressed I–V is marked with the blue and red colors.

There are two main causes for the abnormal hump behavior owing to the device driving stress. The first is the constant voltage/current driving stress applied at the gate electrode, which is similar in character to that of the pixel driving transistors in the OLED display. Figure 2 shows various I–V characteristics (gate voltage sweep from −20 V to 20 V, shown from −5 V to 20 V, under V_ds_ of 0.1 V) as a function of DC stress conditions at 60 °C. The stress was applied under three different bias voltage conditions (V_gs_ = 20 V, V_ds_ = 0.1 V in Fig. 2a; V_gs_ = 40 V, V_ds_ = 20 V in Fig. 2b; and V_gs_ = 20 V, V_ds_ = 40 V in Fig. 2c) for 1.5 h. At 20 V of gate with 0.1 V of drain, the stressed I–V transfer characteristic shows similar behavior to the initial curve; no hump behavior was observed. However, it only shows the shift of the I–V characteristic curves in the stressed one in Fig. 2a. The positive shift of the transfer curve is already well known as trapped charge (*Q*_*trap*_) in the gate insulator and its interface with the active layer,

1 $${Q}_{trap}\cong -\,q\int {n}_{trap}(x)dx$$

with *n*_*trap*_ as the number of traps^19^. In the case of an increased current stress condition, by increasing either the gate voltage or drain voltage, the transfer characteristics of the I–V sweep show the abnormal hump behavior in both conditions, 20 V of drain with 40 V of gate and 40 V of gate with 20 V of drain, as shown in Fig. 2b,c, respectively. The positive shift in the I–V measurement has been increased owing to the increase in the evaluation voltage, and unlike the parallel positive shift only observed in Fig. 2a, the hump characteristics are clearly observed at the V_ds_ sweep measurement together with the current drop at the threshold voltage region, as shown in Fig. 2b,c. To clarify and compare the current drops at the threshold voltage region by the hump depending on the stresses, the I–V characteristic curves were compared through the threshold voltage parallel shift of the initial I–V curves to the stressed data. The initial curve shown in Fig. 2d is the parallel shifted data for the gate voltage of the initial data shown in Fig. 2a. Unlike what is shown in Fig. 2a, a small current drop at the threshold region is observed in Fig. 2d, highlighted by the filled blue color between the shifted initial data and the stressed one. This small reduction in the current is estimated to be caused by increasing defect states near the Fermi level during the stress application, which can be identified as a slight increase in the sub-threshold swing (SS) value from 0.64 V/dec. to 0.66 V/dec. Figure 2e,f show how seriously the current capability is reduced and how seriously the hump occurs in the oxide semiconductor in this DCVS test.

**Figure 3:**
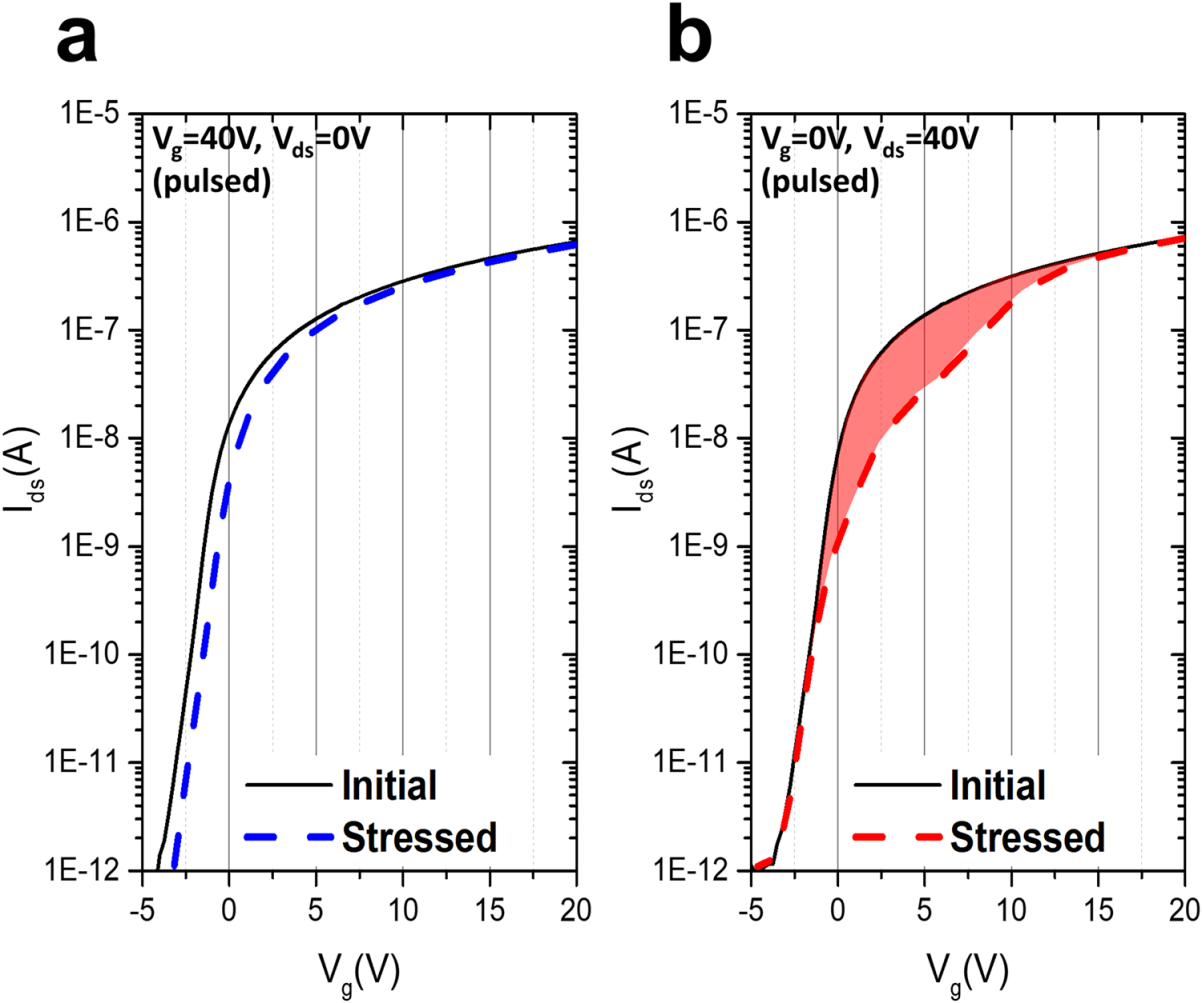
Device characteristics initially and after pulsed high-voltage stress was applied. The black solid line indicates the initial I–V characteristics under V_ds_ = 0.1 V. The blue and red dashed lines are the I–V sweep signals after the stress. (**a**) I–V characteristics after the 1 kHz square-type pulsed signal (duty rate 10% and 40 V amplitude) at the gate side. (**b**) I–V characteristics after the 1 kHz square type pulsed signal (duty rate 10% and 40 V amplitude) at the drain side. The gap between the initial shifted and stressed I–V characteristic curve is marked with filled red color.

The second cause of hump appearance is the pulsed high voltage bias stress applied at the drain electrode, which is similar in character to the gate drive integrated circuit^26^ in display devices. Figure 3 shows the I–V characteristics with 0.1 V_ds_ under the 1 kHz square-type pulsed signal (duty rate 10% and 40 V amplitude). In the case of the pulsed-HVGS, a small shift in the I–V transfer curve is observed in Fig. 3a. The cause of the small positive shift in the threshold voltage is assumed to be the small duty rate, which is 10% of the pulsed signal, and exhibits similar electrical behavior as in Fig. 2a (20 V of gate constant voltage stress). Meanwhile, it was found that the current near the threshold voltage noticeably decreased under the pulsed-HVDS test in Fig. 3b. Figure 3b shows the I–V sweep measurement^3^ after the HVDS test; there was no parallel shift in the I–V curve when compared with the stressed curve. No positive shift was observed, even when 40 V of pulsed signal was applied to the drain. A serious current drop is observed in Fig. 3b, which is similar to the behavior of the current capability degradation shown in Fig. 2f. It was confirmed that the degradation and hump behaviors caused by high current flows (in Fig. 2e: V_gs_ = 20 V and V_ds_ = 40 V; in Fig. 2f: V_gs_ = 40 V, V_ds_ = 20 V) are similar to those caused by a pulsed voltage (V_ds_ = 40 V with 1 kHz under 10% duty, V_gs_ = 0). The amount of current drop caused by the hump increased with stress time and voltage.

### Driving current drop by additional shallow “needle” defects

**Figure 4:**
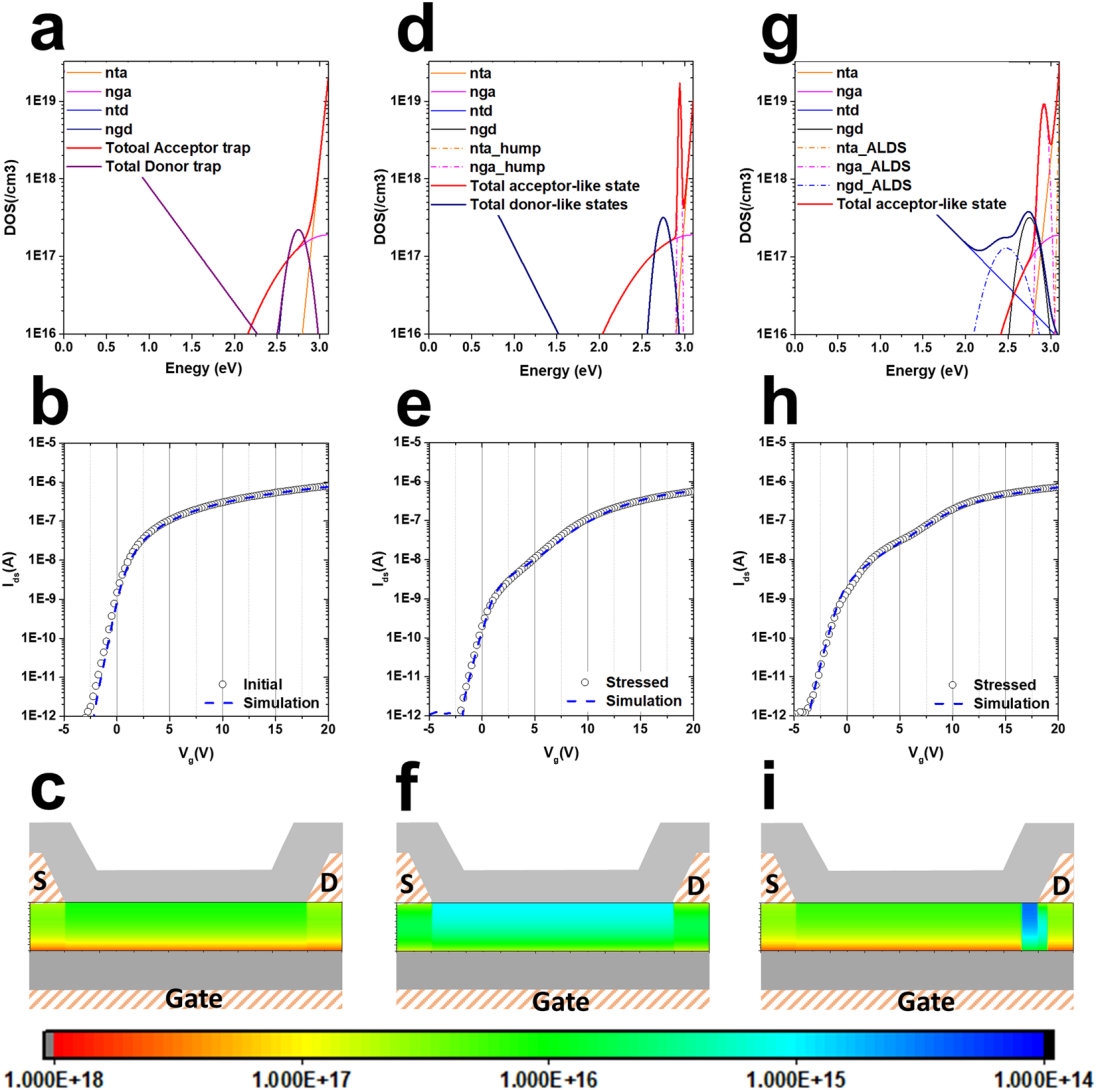
TCAD device simulation by adding sub-gap states. (**a**) Density of the state of the pristine device and (**b**) I–V characteristics of initial experimental and simulated data. *nta*, *nga*, *ntd*, and *ngd* are the number of acceptor-like tail states, acceptor-like Gaussian states, donor-like tailing states, and donor-like Gaussian states, respectively. (**c**) Electron concentration of the pristine device at V_ds_ = 0.1 V with V_g_ = 5 V. After application of the constant bias stress, (**d**) the density of states was extracted and (**e**) simulated (dashed line) the I–V characteristic; (**f**) 2-dimensional electron concentration distribution. (**g**) Density of state for the pulsed-HVDS device and (**h**) the I–V sweep is simulated (dashed line) and compared with experimental data (open circle). An asymmetrical local defect state (ALDS) is located at the edge of the drain electron and (**i**) the electron concentration is disturbed by the ALDS. The rainbow bar tape is the scale bar for electron concentration.

To understand the anomalous hump phenomena in Figs 2 and 3, a 2D numerical TCAD Atlas simulation method was used to calculate the device characteristics. Figure 4a,b show the density of the states and the measured and calculated I–V characteristics for the samples before application of the stress, respectively. The sample not damaged by the stress test does not show the current drop, hump, or abnormal characteristics. This characteristic I–V curve is able to get through the well-known density of states, which is composed of the state position energy, number of states, and width of the states. The donor-like Gaussian states and the acceptor-like Gaussian states in Fig. 4a are positioned close to the CBM. Figure 4c shows the electron concentration at V_ds_ = 0.1 V with V_g_ = 5 V. The more electrons are accumulated at the bottom of the channel owing to the gate electric field, the less electron density is shown under the source/drain electrode.

The case of the damaged sample by DCVS shown in Fig. 2 reveals the serious reduction in the current ability with hump characteristics (Fig. 4e). As shown in Fig. 4d, the density of states when the device is damaged by DC current driving stress is quite different from that of the pristine device. The additional “*needle*” defect state is generated at the nearby CBM, which has a very narrow energy width, 0.016 eV, and high density of states, 1.7 × 10^19^ cm^−3^ eV^−1^. In this study, the additional defect state is the acceptor-like Gaussian state, and it was well matched with experimental data (Fig. 4e). Owing to the additional defect states, the electron distribution density changed, as shown in Fig. 4f. The electron concentration decreased by one order of magnitude compared with that of the pristine device. If a pulse-type signal similar to the operating environment of the gate drive circuit is applied, the characteristic curve shown in Fig. 4h could be obtained.

In the case of the sample damaged from HVDS, the damaged area should be specified on the active channel. When the drain pulse is applied momentarily, the edge of the drain electrode suffers a local damage^20^. In this study, the asymmetrical local defect state (ALDS) was introduced at the edge of the drain electrode only—the resultant density of states is shown in Fig. 4g. The density of state for the ALDS should consider both the hump characteristic, which has a narrow defect state energy width and a high density of acceptor-like Gaussian states near the CBM, and the additional donor-like Gaussian broad state located 0.62 eV away from the CBM. Figure 4i shows the electron concentration of the pulse-type HVDS applied device at V_ds_ = 0.1 V under V_g_ = 5 V. The electron concentration of the channel is similar to that of the pristine device (Fig. 4c), but the concentration at the drain edge shows an extremely low value. It is also possible to verify that the current ability of the entire device is impaired by the damage in an asymmetrical local area rather than in the whole channel region.

**Figure 5:**
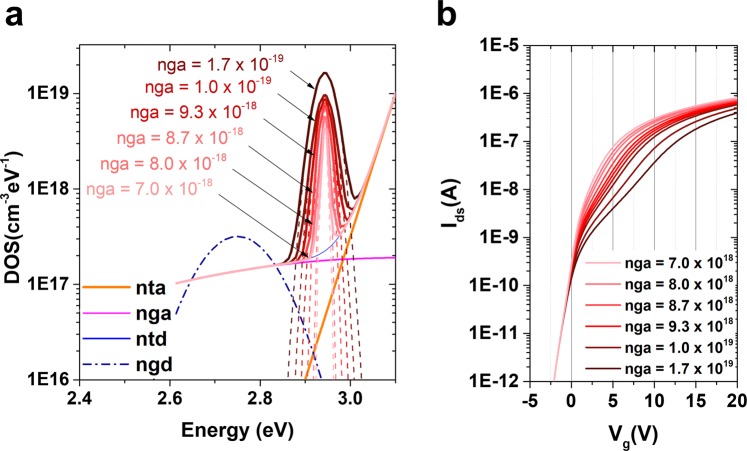
Growth of the acceptor-like Gaussian state. (**a**) Two-dimensional Atlas simulation results as a function of the acceptor-like Gaussian state (cm^−3^ eV^−1^) and energy width of Gaussian (eV). As the defect state increases from 7 × 10^18^ cm^−3^ eV^−1^ with 0.008 eV to 1.7 × 10^19^ cm^−3^ eV^−1^ with 0.03 eV, (**b**) the hump phenomena in the I–V characteristic curve at V_ds_ = 0.1 V become apparent.

The density of states consist largely of four different types of states: acceptor-like Gaussian, donor-like Gaussian, acceptor-like tail, and donor-like tail. Figure 4a shows the four possible states from the normal device, and the defects generated by the hump are also presented in Fig. 4d. It was confirmed that the hump occurred because of the additional generation of an acceptor-like Gaussian state, and if the width of the acceptor-like Gaussian state increases to more than 0.1 eV, no hump is formed. In Fig. 5, this is illustrated in terms of a defect that grows when the stress persists. As time continues in the reliability test, the reduction in current ability due to the hump phenomenon will reach a serious level. This increase in the hump phenomenon could be interpreted as an increase in the additionally generated acceptor-like Gaussian state under the CBM, as shown in Fig. 5a. The growth of the defect is accompanied by an increase in the acceptor-like state and by its width. Figure 5b shows the I–V characteristics as a function of increasing acceptor-like Gaussian state. The increase in the defect explicates the two different shoulders in the hump, at V_g_ = 2.5 V and 10 V.

### Conditions of occurrence of hump phenomenon

**Figure 6:**
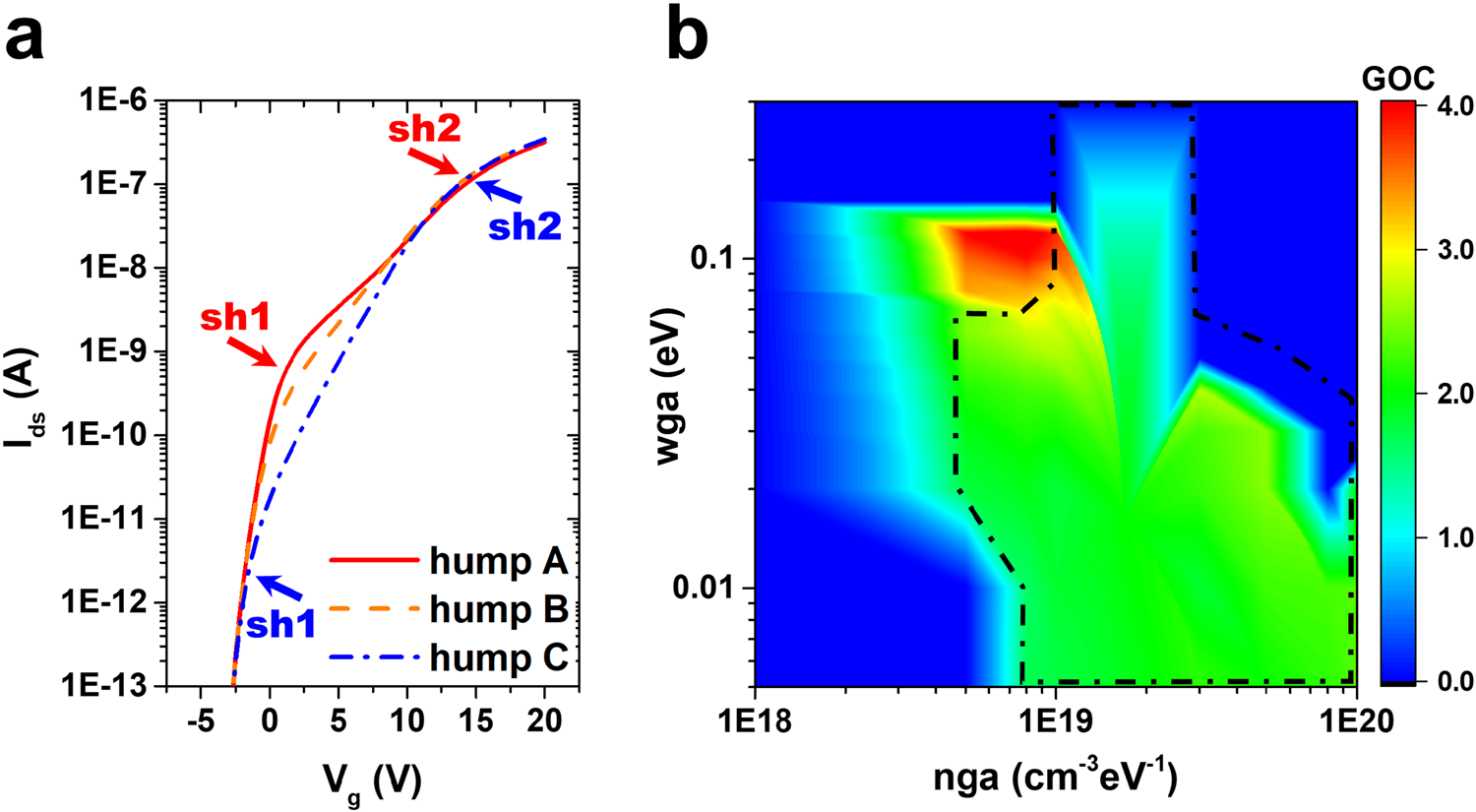
Map of key parameters for hump phenomenon. (**a**) I–V characteristics as function of the *nga* and *wga* parameters; hump A = 8 × 10^19^ cm^−3^ eV^−1^ and 0.005 eV, hump B = 1 × 10^19^ cm^−3^ eV^−1^ and 0.06 eV, and hump C = 5 × 10^18^ cm^−3^ eV^−1^ and 0.12 eV. *sh1* and *sh2* indicate the current of the first and second shoulders in the hump, respectively. (**b**) Gap of current (GOC) between *sh1* and *sh2* as a function of the number of acceptor-like Gaussian defects (*nga*) and their width (*wga*). The dashed red line indicates the borderline greater than 10^−10^ A of *sh1*. In the defect position, the energy level of the *nga* is fixed as 0.157 eV.

In the evaluation of the device characteristics, it was found that the hump phenomenon occurs only when the defect state appears in a specific location with a specific energy. Before the discussion of the conditions, here we define “*sh1*” and “*sh2*” to identify the hump characteristics. “*sh1*” is specified by the drain current level at the first shoulder of the hump curve, where the curve moves away from the initial curve. “*sh2*” is specified by the drain current at the second shoulder of the hump curves at high gate voltage (above V_g_ = 10 V). In Fig. 6a, “hump A” shows clearer hump characteristics than “hump C,” which depends on the *wga* and *nga* values. Figure 6a depicts the *wga* dependence and the “*sh1*” value is reduced with increasing *wga*. The *wga* values of humps A, B, and C are 0.005, 0.06, and 0.12 eV, respectively. Figure 6b illustrates the conditions under which a hump could occur in the relation between number of acceptor-like Gaussian defect states (*nga*) and width of the energy (*wga*). The position energy of the acceptor-like Gaussian states is fixed as 0.157 eV, which is the same as the value shown in Fig. 4. The displayed “gap of current” (GOC) is defined as “*sh1*” and “*sh2*,” where the hump occurs in any I–V characteristic graph, indicating the difference in current value at the point where the hump occurs.

2 $${\rm{GOC}}\equiv \,\mathrm{log}\,(sh2)-\,\mathrm{log}(sh1)$$

For the humps generated in the instability test, the GOC value is mostly close to two, similar to “hump A,” as illustrated in Fig. 6a ^9,15,18,27,28^. Of particular importance is the fact that the current value of the first hump shoulder, which is the assigned *sh1* reading, occurs between 10^−9^ A and 10^−10^ A. Although the current value of “*sh1*” may vary depending on the type of defect that occurs in the device, many studies report a figure greater than 10^−10^ A in the V_ds_ = 0.1 V sweep. The *sh1* value greater than 10^−10^ A is indicated as the black dashed line in Fig. 6b. This study does not take into account “hump C” (where the *sh1* value is lower than 10^−11^ A in the V_ds_ = 0.1 V sweep) that could be produced in the simulation but is difficult to generate in the instability experiment.

### Hump by predictable edge current

**Figure 7:**
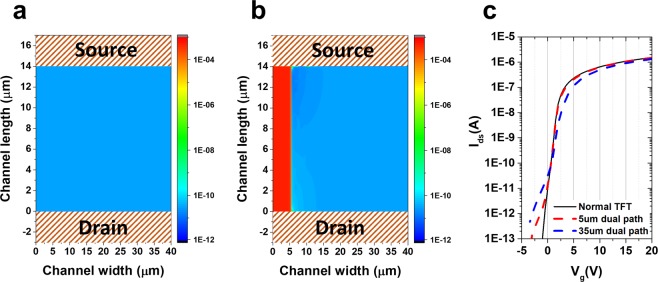
Edge current effect in the TFT. Two-dimensional cut-plane (plane at 0.1 nm away from the top of the active layer) of the current density of (**a**) the normal device and (**b**) the device with the conductive channel edge under V_gs_ = −2 V and V_ds_ = 0.1 V on the active layer from the 3D device simulation. (**c**) I–V characteristics of the TFT without dual current path and dual path TFT with 5 μm and 35 μm conducting additional current paths in the active channel layer.

Up to this point, the discussion has been centered on the generation or increase of the defect states in the density of states as the cause of the hump. The occurrence of the channel edge current could not be ignored owing to the various issues and causes of the hump device. To evaluate the effect of the edge current, possible phenomena were identified in the presence of a conductive area in the active edge region using the 3D Atlas TCAD simulation. Figure 7a,b present, from the 3D device simulation, the total current density of the cut-plane of 39.9 nm in channel thickness of the normal device and the device with the conductive channel edge on the active layer, respectively. If contamination or local deformation occurs owing to various issues on the edge of the device, the edge current would be higher than the current in the entire channel area, as shown in Fig. 7b, resulting in more than one current path. This parasitic current path is shorter than the width of the device, and is characterized by higher electron concentration than that of the surrounding path. In the event of parasitic current, the characteristic I-V curves of the devices (normal TFT without dual current path and dual channel TFT with 5 𝜇m and 35 𝜇m conducting current path in the channel) could be formed, as shown in Fig. 7c, and the hump characteristic could be determined as expected. However, in the case of hump generation due to edge current, the “*sh1*” value is formed too low, and the form of the depredated characteristic curves differs from the measured data in Figs 2 and 3.

## Discussion

In this paper, we report on the origin of different drops of current ability with the hump phenomenon. There are two main causes for the degradation with abnormal hump behavior due to the device driving stress. The first cause is the constant voltage/current driving stress applied at the gate electrode, which is similar in character to that of the pixel driving transistors in OLED displays and electronic devices. The second cause of hump appearance is the pulsed high voltage bias stress applied at the drain electrode, which is similar in character to that of the gate drive integrated circuit. It was confirmed that the reduction in the driving current ability accompanied by the hump is caused by the generation of acceptor-like Gaussian needle defect states, which is a narrow (<0.1 eV) width of the Gaussian, located at 0.157 eV from the CBM. In particular, the ALDS demonstrated that it induces a reduction in current by further generation of the donor-like Gaussian defect state and acceptor-like Gaussian defect states at the bottom of the drain electrode edge. The hump phenomenon occurs because of the formation of the *nga* with the *wga* in the additional acceptor-like defect state. However, if the *nga* develops larger than a certain size, the hump phenomenon does not appear and only the current drop could be shown. If a dual current path is present in the channel area owing to the occurrence of edge current, the hump phenomenon would occur, but it would not have behaviors similar to the I–V characteristic curves reported in the experiment.

Reliability-related hump issues have existed for decades, as many papers on oxide semiconductors presented over the years can attest. Since the development of the oxide semiconductor, extensive research has been conducted on the threshold voltage shift caused by gate charging and the state generation on the interface of the gate insulator. Regarding reliability, a reduction in the current ability accompanied by a hump would degrade the device’s ability to operate and cause serious problems in the long-term reliability of the electronic product in the field. In this study, it was clarified that the reduction in current ability caused by the high voltage and AC driving is as a result of the narrow and sharp additional acceptor-like Gaussian defect state on the underside of the CBM. The results of this study are expected to serve as a basis for further research aimed at reducing the instability of the oxide semiconductor and expanding its applications in different devices.

## Methods

### Device fabrication

The *a*-IGZO TFTs were fabricated on an un-doped Si wafer. Mo metal was used as the gate electrode, which was deposited by sputtering and patterned by photolithography and wet etching, and its defined width was 20 μm. The gate insulator layer was composed of SiO_x_ and SiN_x_ dual layers by plasma-enhanced chemical vapor deposition (PECVD) at 350 °C. A 40-nm *a*-IGZO film was sputtered by RF sputtering (4-inch target, with a distance of 120 mm between the source and the substrate, Korea Vacuum Tech, Inc.) at 100 °C using IGZO_x_ ceramic target (In:Ga:Zn = 1:1:1 atomic ratio) under a gas mixture of Ar and O_2_ and an input power of 200 W (5 mTorr). The active layer was defined by photolithography. Mo metal was used as the source/drain electrode, and the electrode was defined (width of 40 μm and length of 14 μm) by the lift-off technique on the active layer. It was designed with 3 μm of overlap between the source/drain and gate electrode. The gate-via was formed by a reactive ion dry etching process. A passivation layer (SiO_x_, 200 nm) was formed by PECVD on the *a*-IGZO TFTs and it was annealed at 350 °C for 1 h in a vacuum environment to improve the contact and stability of the transistors.

### Measurement

Current vs. voltage (I–V) measurement (gate voltage sweep from −20 V to 20 V, shown from −5 V to 20 V) was carried out under V_ds_ = 0.1 V using the Keithley 2636B. Two different electric stresses were applied at 60 °C. The first was a constant gate and drain DC voltage (V_gs_ = 20 V, V_ds_ = 40 V or V_gs_ = 40 V, V_ds_ = 20 V). The second, a pulse-type high voltage at the drain side (HVDS) and high voltage at the gate side (HVGS) stress, was applied (1 kHz frequency, duty cycle 10%, pulse rising/fall time 1 × 10^−8^ s, drain turn on voltage +40 V, turn off voltage 0 V @ *V*_*g*_ = 0 V) using Agilent 81160A and high speed bipolar amplifier HDS 4011. The electrical stress was applied for 1.5 h.

### Atlas TCAD simulation

A device simulator TCAD was used in this study to understand the electron transport properties. The simulation was conducted using the Silvaco’s 2D and 3D ATLAS simulator package^22^. A configuration similar to that of the fabricated materials and devices was employed by the Athena fabrication simulator. For the simulation, the following characteristic parameters were used as input values: the relative permittivity of the silicon oxide as the gate insulator and *a*-IGZO as the channel semiconductor was 3.9 and 13, respectively; the electron affinity of *a*-IGZO was 4.1 eV; the band gap and mobility were 3.1 eV and 9.5 cm^2^V^−1^s^−1^, respectively; the effective densities of states in the conduction and valence bands were 5.0 × 10^18^ cm^−3^ and 4.6 × 10^19^ cm^−3^, respectively.

## Data Availability

The datasets generated during and/or analyzed during the current study are available from the corresponding author on reasonable request.

## References

[1] Franklin A. D. (2015). Nanomaterials in transistors: From high-performance to thin-film applications. Science.

[2] Conley JF (2010). Instabilities in Amorphous Oxide Semiconductor Thin-Film Transistors. IEEE Transactions on Device and Materials Reliability.

[3] Lee H-J, Cho SH, Abe K, Lee M-J, Jung M (2017). Impact of transient currents caused by alternating drain stress in oxide semiconductors. Scientific Reports.

[4] Suresh A, Muth JF (2008). Bias stress stability of indium gallium zinc oxide channel based transparent thin film transistors. Appl. Phys. Lett..

[5] Nomura K, Kamiya T, Hirano M, Hosono H (2009). Origins of threshold voltage shifts in room-temperature deposited and annealed a-In–Ga–Zn–O thin-film transistors. Appl. Phys. Lett..

[6] Jeong JK, Yang HW, Jeong JH, Mo Y-G, Kim HD (2008). Origin of threshold voltage instability in indium-gallium-zinc oxide thin film transistors. Appl. Phys. Lett..

[7] Tsai M-Y (2013). Asymmetric structure-induced hot-electron injection under hot-carrier stress in a-InGaZnO thin film transistor. Appl. Phys. Lett..

[8] Cho Y-J (2017). Effect of illumination on the hump phenomenon in I–V characteristics of amorphous InGaZnO TFTs under positive gate-bias stress. physica status solidi (a).

[9] Lee H-J (2018). Drain-Induced Barrier Lowering in Oxide Semiconductor Thin-Film Transistors With Asymmetrical Local Density of States. IEEE Journal of the Electron Devices Society.

[10] Jeong C-Y (2014). A study on the degradation mechanism of InGaZnO thin-film transistors under simultaneous gate and drain bias stresses based on the electronic trap characterization. Semiconductor Science and Technology.

[11] Tsao Y-C (2017). Abnormal hump in capacitance–voltage measurements induced by ultraviolet light in a-IGZO thin-film transistors. Appl. Phys. Lett..

[12] Valletta A, Gaucci P, Mariucci L, Fortunato G, Templier F (2008). “Hump” characteristics and edge effects in polysilicon thin film transistors. Journal of Applied Physics.

[13] Sallagoity P, Ada-Hanifi M, Paoli M, Haond M (1996). Analysis of width edge effects in advanced isolation schemes for deep submicron CMOS technologies. IEEE Transactions on Electron Devices.

[14] Huang CF (2008). Stress-Induced Hump Effects of p-Channel Polycrystalline Silicon Thin-Film Transistors. IEEE Electron Device Letters.

[15] Hwarim I, Hyunsoo S, Jaewook J, Yewon H, Yongtaek H (2015). Effects of defect creation on bidirectional behavior with hump characteristics of InGaZnO TFTs under bias and thermal stress. Japanese Journal of Applied Physics.

[16] Tsai M-Y (2013). High temperature-induced abnormal suppression of sub-threshold swing and on-current degradations under hot-carrier stress in a-InGaZnO thin film transistors. Appl. Phys. Lett..

[17] Liu PT (2018). Highly Responsive Blue Light Sensor with Amorphous Indium-Zinc-Oxide Thin-Film Transistor based Architecture. Scientific Reports.

[18] Mativenga M, Seok M, Jang J (2011). Gate bias-stress induced hump-effect in transfer characteristics of amorphous-indium-galium-zinc-oxide thin-fim transistors with various channel widths. Appl. Phys. Lett..

[19] Lee J (2015). Modeling and Characterization of the Abnormal Hump in n-Channel Amorphous-InGaZnO Thin-Film Transistors After High Positive Bias Stress. IEEE Electron Device Letters.

[20] Lee H-J, Abe K, Kim JS, Lee M-J (2017). Electron-blocking by the potential barrier originated from the asymmetrical local density of state in the oxide semiconductor. Scientific Reports.

[21] Wang Dapeng, Zhao Wenjing, Li Hua, Furuta Mamoru (2018). Drain Current Stress-Induced Instability in Amorphous InGaZnO Thin-Film Transistors with Different Active Layer Thicknesses. Materials.

[22] ATLAS Device Simulation Software User’s Manual. *Silvaco International, Santa Clara, CA* (2018).

[23] Ryu SH, Park YC, Mativenga M, Kang DH, Jang J (2012). Amorphous-InGaZnO4 Thin-Film Transistors with Damage-Free Back Channel Wet-Etch Process. ECS Solid State Letters.

[24] Kim M (2007). High mobility bottom gate InGaZnO thin film transistors with SiOx etch stopper. Appl. Phys. Lett..

[25] Yang J (2014). A Short-Channel TFT of Amorphous In–Ga–Zn–O Semiconductor Pixel Structure With Advanced Five-Mask Process. IEEE Electron Device Letters.

[26] Takeshi O (2010). Development of Liquid Crystal Display Panel Integrated with Drivers Using Amorphous In–Ga–Zn-Oxide Thin Film Transistors. Japanese Journal of Applied Physics.

[27] Yang J (2017). Investigation of an anomalous hump phenomenon in via-type amorphous In-Ga-Zn-O thin-film transistors under positive bias temperature stress. Appl. Phys. Lett..

[28] Shin Y (2017). The Mobility Enhancement of Indium Gallium Zinc Oxide Transistors via Low-temperature Crystallization using a Tantalum Catalytic Layer. Scientific Reports.

